# Supplementary material for: Modeling glioblastoma heterogeneity as a dynamic network of cell states
Source: Mol Syst Biol. 2021 Sep 16;17(9):e10105. doi: 10.15252/msb.202010105 (PMC8444284; doi:10.15252/msb.202010105)
Supplement: Supplementary file 5 — Source Data for Figure 3 [file MSB-17-e10105-s001.zip › Figure3A_sourcedata/GSEA_3065/hallmarks_state3.GseaPreranked.1623416274744/HALLMARK_ESTROGEN_RESPONSE_EARLY.html]

Details for gene set HALLMARK\_ESTROGEN\_RESPONSE\_EARLY[GSEA]

|  || Dataset | state3 |
| Phenotype | NoPhenotypeAvailable |
| Upregulated in class | na\_pos |
| GeneSet | HALLMARK\_ESTROGEN\_RESPONSE\_EARLY |
| Enrichment Score (ES) | 0.33926868 |
| Normalized Enrichment Score (NES) | 1.3134061 |
| Nominal p-value | 0.05706522 |
| FDR q-value | 0.13459432 |
| FWER p-Value | 0.666 |
Table: GSEA Results Summary

  

Fig 1: Enrichment plot: HALLMARK\_ESTROGEN\_RESPONSE\_EARLY      
 Profile of the Running ES Score & Positions of GeneSet Members on the Rank Ordered List

  

| PROBE | GENE SYMBOL | GENE\_TITLE | RANK IN GENE LIST | RANK METRIC SCORE | RUNNING ES | CORE ENRICHMENT || 1 | ABHD2 |  |  | 36 | 0.467 | 0.0453 | Yes |
| 2 | SLC16A1 |  |  | 138 | 0.314 | 0.0670 | Yes |
| 3 | PODXL |  |  | 314 | 0.240 | 0.0722 | Yes |
| 4 | IL6ST |  |  | 316 | 0.239 | 0.0975 | Yes |
| 5 | SLC26A2 |  |  | 442 | 0.210 | 0.1053 | Yes |
| 6 | ELOVL5 |  |  | 450 | 0.209 | 0.1267 | Yes |
| 7 | NAV2 |  |  | 486 | 0.201 | 0.1439 | Yes |
| 8 | FASN |  |  | 503 | 0.198 | 0.1631 | Yes |
| 9 | SLC39A6 |  |  | 512 | 0.197 | 0.1831 | Yes |
| 10 | SLC2A1 |  |  | 587 | 0.188 | 0.1944 | Yes |
| 11 | ENDOD1 |  |  | 623 | 0.184 | 0.2099 | Yes |
| 12 | DHCR7 |  |  | 669 | 0.176 | 0.2233 | Yes |
| 13 | LRIG1 |  |  | 772 | 0.161 | 0.2286 | Yes |
| 14 | GLA |  |  | 816 | 0.156 | 0.2401 | Yes |
| 15 | TUBB2B |  |  | 904 | 0.148 | 0.2458 | Yes |
| 16 | RHOBTB3 |  |  | 944 | 0.143 | 0.2565 | Yes |
| 17 | SEMA3B |  |  | 956 | 0.142 | 0.2702 | Yes |
| 18 | XBP1 |  |  | 1036 | 0.135 | 0.2754 | Yes |
| 19 | CELSR2 |  |  | 1135 | 0.127 | 0.2775 | Yes |
| 20 | GJA1 |  |  | 1160 | 0.125 | 0.2880 | Yes |
| 21 | FLNB |  |  | 1335 | 0.113 | 0.2799 | Yes |
| 22 | SLC7A5 |  |  | 1394 | 0.109 | 0.2847 | Yes |
| 23 | RETREG1 |  |  | 1395 | 0.109 | 0.2962 | Yes |
| 24 | HES1 |  |  | 1501 | 0.103 | 0.2950 | Yes |
| 25 | UGCG |  |  | 1551 | 0.101 | 0.2999 | Yes |
| 26 | MYBBP1A |  |  | 1686 | 0.093 | 0.2943 | Yes |
| 27 | CA12 |  |  | 1739 | 0.089 | 0.2977 | Yes |
| 28 | CD44 |  |  | 1748 | 0.089 | 0.3062 | Yes |
| 29 | NCOR2 |  |  | 1781 | 0.087 | 0.3118 | Yes |
| 30 | SCARB1 |  |  | 1789 | 0.087 | 0.3203 | Yes |
| 31 | OLFM1 |  |  | 1809 | 0.086 | 0.3272 | Yes |
| 32 | PRSS23 |  |  | 1890 | 0.081 | 0.3266 | Yes |
| 33 | WFS1 |  |  | 1891 | 0.081 | 0.3352 | Yes |
| 34 | SLC1A4 |  |  | 2003 | 0.076 | 0.3304 | Yes |
| 35 | IGF1R |  |  | 2037 | 0.074 | 0.3345 | Yes |
| 36 | ABCA3 |  |  | 2201 | 0.068 | 0.3229 | Yes |
| 37 | AKAP1 |  |  | 2203 | 0.068 | 0.3300 | Yes |
| 38 | MED13L |  |  | 2228 | 0.067 | 0.3343 | Yes |
| 39 | AR |  |  | 2247 | 0.066 | 0.3393 | Yes |
| 40 | MYC |  |  | 2324 | 0.064 | 0.3372 | No |
| 41 | AFF1 |  |  | 2735 | 0.049 | 0.2951 | No |
| 42 | CANT1 |  |  | 2901 | 0.044 | 0.2807 | No |
| 43 | ADD3 |  |  | 2929 | 0.043 | 0.2821 | No |
| 44 | REEP1 |  |  | 2960 | 0.042 | 0.2831 | No |
| 45 | MYOF |  |  | 2989 | 0.041 | 0.2842 | No |
| 46 | B4GALT1 |  |  | 3043 | 0.040 | 0.2824 | No |
| 47 | ABAT |  |  | 3059 | 0.040 | 0.2848 | No |
| 48 | NRIP1 |  |  | 3312 | 0.033 | 0.2592 | No |
| 49 | MAPT |  |  | 3333 | 0.032 | 0.2603 | No |
| 50 | PDLIM3 |  |  | 3422 | 0.030 | 0.2533 | No |
| 51 | STC2 |  |  | 3529 | 0.027 | 0.2440 | No |
| 52 | FARP1 |  |  | 3580 | 0.026 | 0.2409 | No |
| 53 | TSKU |  |  | 3587 | 0.026 | 0.2430 | No |
| 54 | SIAH2 |  |  | 3635 | 0.025 | 0.2402 | No |
| 55 | FOS |  |  | 3899 | 0.018 | 0.2117 | No |
| 56 | ISG20L2 |  |  | 3955 | 0.016 | 0.2070 | No |
| 57 | ADCY9 |  |  | 4036 | 0.014 | 0.1993 | No |
| 58 | FKBP5 |  |  | 4089 | 0.013 | 0.1947 | No |
| 59 | AMFR |  |  | 4305 | 0.007 | 0.1705 | No |
| 60 | ELF1 |  |  | 4377 | 0.004 | 0.1628 | No |
| 61 | NADSYN1 |  |  | 4437 | 0.003 | 0.1563 | No |
| 62 | SEC14L2 |  |  | 4554 | -0.000 | 0.1429 | No |
| 63 | KDM4B |  |  | 4739 | -0.004 | 0.1221 | No |
| 64 | CELSR1 |  |  | 4969 | -0.010 | 0.0967 | No |
| 65 | TPBG |  |  | 5003 | -0.011 | 0.0941 | No |
| 66 | CXCL12 |  |  | 5164 | -0.015 | 0.0772 | No |
| 67 | BHLHE40 |  |  | 5331 | -0.019 | 0.0600 | No |
| 68 | SYNGR1 |  |  | 5397 | -0.021 | 0.0547 | No |
| 69 | KLF10 |  |  | 5514 | -0.025 | 0.0439 | No |
| 70 | MED24 |  |  | 5537 | -0.025 | 0.0440 | No |
| 71 | WWC1 |  |  | 5860 | -0.035 | 0.0105 | No |
| 72 | TIAM1 |  |  | 5861 | -0.035 | 0.0142 | No |
| 73 | RPS6KA2 |  |  | 6074 | -0.042 | -0.0059 | No |
| 74 | SVIL |  |  | 6089 | -0.042 | -0.0030 | No |
| 75 | FKBP4 |  |  | 6339 | -0.051 | -0.0264 | No |
| 76 | TFAP2C |  |  | 6436 | -0.054 | -0.0318 | No |
| 77 | ITPK1 |  |  | 6474 | -0.055 | -0.0302 | No |
| 78 | UNC119 |  |  | 6557 | -0.059 | -0.0334 | No |
| 79 | FDFT1 |  |  | 6637 | -0.063 | -0.0359 | No |
| 80 | MREG |  |  | 6671 | -0.064 | -0.0328 | No |
| 81 | FAM102A |  |  | 6807 | -0.071 | -0.0409 | No |
| 82 | INPP5F |  |  | 6837 | -0.072 | -0.0366 | No |
| 83 | PPIF |  |  | 6999 | -0.081 | -0.0466 | No |
| 84 | TIPARP |  |  | 7051 | -0.084 | -0.0436 | No |
| 85 | TOB1 |  |  | 7088 | -0.086 | -0.0386 | No |
| 86 | RBBP8 |  |  | 7128 | -0.089 | -0.0337 | No |
| 87 | FHL2 |  |  | 7163 | -0.091 | -0.0280 | No |
| 88 | NBL1 |  |  | 7258 | -0.097 | -0.0285 | No |
| 89 | BAG1 |  |  | 7323 | -0.102 | -0.0251 | No |
| 90 | NXT1 |  |  | 7422 | -0.110 | -0.0248 | No |
| 91 | SNX24 |  |  | 7428 | -0.110 | -0.0136 | No |
| 92 | SH3BP5 |  |  | 7436 | -0.111 | -0.0026 | No |
| 93 | CCND1 |  |  | 7456 | -0.113 | 0.0071 | No |
| 94 | BLVRB |  |  | 7460 | -0.113 | 0.0188 | No |
| 95 | CHPT1 |  |  | 7521 | -0.119 | 0.0244 | No |
| 96 | ARL3 |  |  | 7744 | -0.145 | 0.0141 | No |
| 97 | PMAIP1 |  |  | 8157 | -0.217 | -0.0105 | No |
| 98 | DYNLT3 |  |  | 8205 | -0.230 | 0.0085 | No |
| 99 | RAB31 |  |  | 8696 | -0.514 | 0.0064 | No |
Table: GSEA details [plain text format]

  

Fig 2: HALLMARK\_ESTROGEN\_RESPONSE\_EARLY: Random ES distribution      
 Gene set null distribution of ES for **HALLMARK\_ESTROGEN\_RESPONSE\_EARLY**
